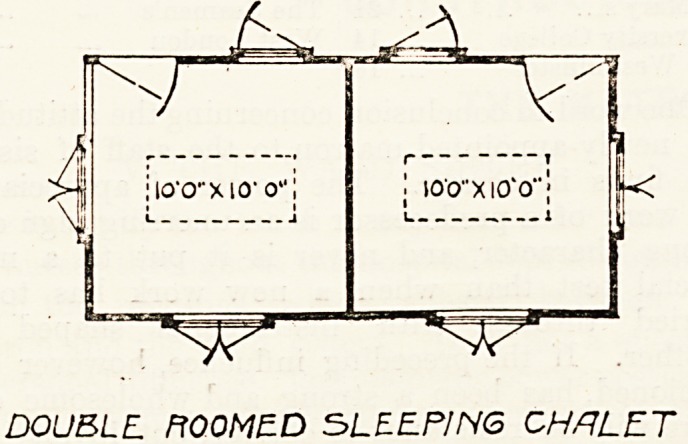# Bellefield Sanatorium for Consumptives, Lanark

**Published:** 1905-09-16

**Authors:** 


### BELLEFIELD SANATORIUM FOR CONSUMPTIVES, LANARK.

After full consideration, the committee appointed to carry out this Sanatorium, decided to erect an inexpensive building, and to conserve as much as possible of the available funds to meet the working expenses. Undoubtedly their decision was a wise one, and to us it has always been a matter of surprise that such a course is not oftener followed more especially in sanatoria for consumptives, as up to the present time no system of construction and no plan of building can be said to have evolved itself into a model for future designers, although the tendency should be, and we think is, towards single-bedded chalets surrounding an administrative centre.

The plan before us is evidently only part of a scheme; the west wing and half of the centre being put up. S far it consists of a day-room having its serving-room, kitchen, and other offices to the north; the day-room itself being exposed to the east and to the south. Stretching westwards are five double-bedded rooms. These face the south, have large bay-

DOUBLE ROOMED SLEEPING CHALET

windows and the doors (with fanlights), are placed opposite and open into a well-lighted corridor, which arrangement ensures good cross-ventilation. Next the most westerly of these double-bedded rooms is the nurse's duty-room; then a corridor which, on one hand, communicates with the ten-bedded ward, and on the other with the sanitary block. Beyond the corridor is the test-room.

The ten-bedded ward has a south-easterly exposure, and it seems to be efficiently lighted and ventilated. Many of the windows are carried out on the French system, opening to the floor level, which not only permits perflation of air in the lower parts of the room but allows the beds to be wheeled on to a cement platform which surrounds the whole front of the Sanatorium. On the grounds are five double-bedded chalets, and these little blocks have each six windows and two doors. It is more than probable that better results will be obtained here than in the wards. The total accommodation is for 30 patients.

The buildings were designed and erected by Messrs. Spiers and Co., of Glasgow, and on a system patented by them, which is said to be only one-third as costly as the ordinary kind of building. Unfortunately the cost of this Sanatorium is not stated.

A house stood on the Bellefield Estate when it was bought by the committee, and this house is being used for the resident staff.

## Figures and Tables

**Figure f1:**
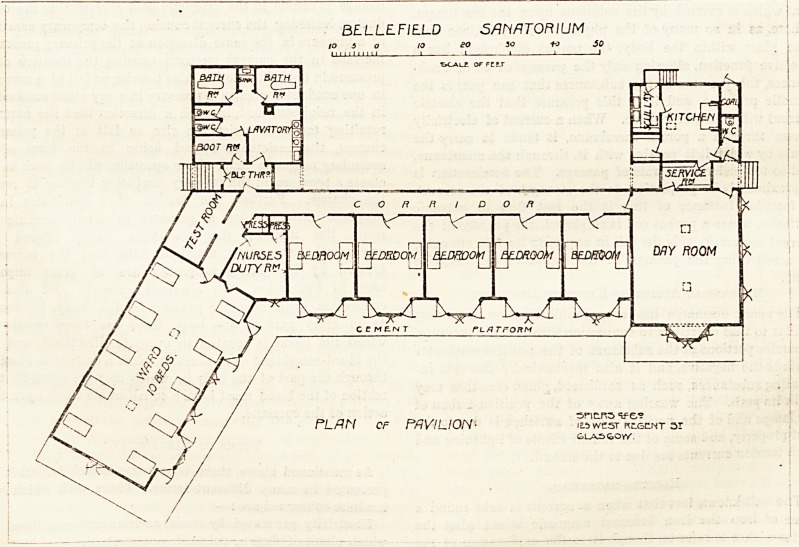


**Figure f2:**